# A latent class analysis of cognitive decline in US adults, BRFSS 2015-2020

**DOI:** 10.1186/s12889-022-14001-2

**Published:** 2022-08-16

**Authors:** Ryan Snead, Levent Dumenci, Resa M. Jones

**Affiliations:** 1 Department of Epidemiology & Biostatistics, Temple University, Philadelphia, Pennsylvania USA; 2 Fox Chase Cancer Center, Temple University Health System, Philadelphia, Pennsylvania USA

**Keywords:** Latent Class Analysis, Dementia, Alzheimer’s, BRFSS, Aging, Subjective Cognitive Decline, Complex Sampling

## Abstract

**Background:**

Cognitive decline can be an early indicator for dementia. Using quantitative methods and national representative survey data, we can monitor the potential burden of disease at the population-level.

**Methods:**

BRFSS is an annual, nationally representative questionnaire in the United States. The optional cognitive decline module is a six-item self-reported scale pertaining to challenges in daily life due to memory loss and growing confusion over the past twelve months. Respondents are 45+, pooled from 2015-2020. Latent class analysis was used to determine unobserved subgroups of subjective cognitive decline (SCD) based on item response patterns. Multinomial logistic regression predicted latent class membership from socio-demographic covariates.

**Results:**

A total of 54,771 reported experiencing SCD. The optimal number of latent classes was three, labeled as Mild, Moderate, and Severe SCD. Thirty-five percent of the sample belonged to the Severe group. Members of this subgroup were significantly less likely to be older (65+ vs. 45-54 OR = 0.29, 95% CI: 0.23-0.35) and more likely to be non-Hispanic Black (OR = 1.80, 95% CI: 1.53-2.11), have not graduated high school (OR = 1.60, 95% CI: 1.34-1.91), or earned <$15K a year (OR = 3.03, 95% CI: 2.43-3.77).

**Conclusions:**

This study determined three latent subgroups indicating severity of SCD and identified socio-demographic predictors. Using a single categorical indicator of SCD severity instead of six separate items improves the versatility of population-level surveillance.

## Background

Dementia, such as Alzheimer’s disease, significantly impact our increasingly aging population. In the US, in 2014 about 5 million people were living with Alzheimer’s disease or a dementia-related disease, which is projected to double by 2060 [1]. Cognitive decline, an early warning sign for dementia, becomes apparent in one’s inability to manage typical daily activities, household chores, and social interactions [2–7]. Overall, the national impact of cognitive decline on daily functioning prompted calls for enhanced surveillance and data collection [8, 9]. As a result, the Centers for Disease Control and Prevention’s (CDC) Healthy Aging program’s Healthy Brain Initiative developed an instrument to measure increased confusion and memory loss. In 2015, the module has been refined to a six-item measure administered in the CDC’s Behavioral Risk Factor Surveillance System (BRFSS) survey to reflect subjective cognitive decline (SCD) [10, 11]. Results from 2019-2020 show that 1 in 10 people 45 years and older are experiencing SCD, improving slightly from 2015-2018 (1 in 9) [12–14]. Since 2015, less than half of people experiencing SCD discussed their symptoms with a healthcare provider [12–14].

The use of the cognitive decline module in BRFSS allows public health professionals and academic researchers to understand the prevalence of SCD at the state and national level. To date, analysis is limited to descriptive statistics of individual items. Unfortunately, this does not distinguish the severity of respondent’s SCD, an important distinction for most effectively providing care to a population. Further, the current structure is difficult to assess differential experiences of cognitive decline predicted by socio-demographics or behavior. Reported response patterns may discern subgroups (or classes) of severity, for example, having moderate difficulty performing daily functions due to cognitive decline. Using quantitative methods, one can assess unobserved (latent) categories of respondents’ item responses. Latent class analysis (LCA) has been used in aging research to understand motives for exercise, cognitive subtypes of people with Alzheimer’s disease, and profiles of cognitive impairment [15–17]. To the best of the authors’ knowledge, there are no studies examining the discrete latent groupings of the BRFSS cognitive decline module or dementia-related national surveillance measures.

While US states now have the ability to annually quantify the burden of declining cognition in their population, the severity of SCD and its impact are not well understood. It is important to consider varying degrees of cognitive decline to aid in planning, resource allocation, and long-term care needs. The purpose of this study is to conduct LCA to investigate unobserved subgroups of SCD in the last year. In addition, the study seeks to assess how socio-demographic covariates are associated with membership of unobserved cognitive decline subgroups. The authors hypothesize that among individuals who are experiencing worsening confusion and memory loss, response patterns will yield discrete latent subgroups representing indicators of severity in respondent SCD. Further, the authors hypothesize that socio-demographic characteristics will predict latent class membership.

## Methods

### Participants and eligibility

From 2015 to 2020, 597,907 noninstitutionalized adults, aged 45+ residing in the US completed the optional BRFSS cognitive decline module. Only respondents who answered “yes” to having experiences of confusion or memory loss occurring more often or getting worse over the past 12 months were included in the analysis (*n*=54,771). Respondents who did not report confusion or memory loss in the past 12 months were excluded (*n*=543,136).

### Behavioral risk factor surveillance system

BRFSS is an annual self-report questionnaire covering chronic disease, preventative services, and health behavior collecting data from all 50 states and US territories [18–20]. BRFSS is telephone-based and reaches respondents using random digit dialing for landlines and cellphones [18–20]. Each state employs complex sampling to account for underrepresented populations [18–20]. The questionnaire consists of a national core component with various health-related measures and demographics, as well as optional modules such as the cognitive decline module which varies state-by-state [18–20]. This study uses pooled BRFSS data from 2015 to 2020 for demographic, behavioral, and cognitive decline variables. If a state administered the cognitive decline module more than once over the six-year period, each iteration was included in the sample. In total, 52 states and territories are represented in the final data set. Only Guam is not present in the sample as the territory has not administered the module over our study period.

### Subjective cognitive decline measure and coding

Five of the six-item SCD questions were used (4 5-category ordinal; 1 binary), assessing self-reported challenges in daily life due to memory loss and growing confusion over the past twelve months. The first question of the module was excluded as this item acts as a screener for skip logic. Only those who reported SCD answered the following questions. Four questions had *Always, Usually, Sometimes, Rarely, and Never* responses, which were: *2) “During the past 12 months, as a result of confusion or memory loss, how often have you given up day-to-day household activities or chores you used to do, such as cooking, cleaning, taking medications, driving, or paying bills?”; 3) “As a result of confusion or memory loss, how often do you need assistance with these day-to-day activities?”; 4)“When you need help with these day-to-day activities, how often are you able to get the help that you need?”;* and *5)“During the past 12 months, how often has confusion or memory loss interfered with your ability to work, volunteer, or engage in social activities outside the home?”* Item 4 was coded as “Never” when item 3 was answered “Rarely” or “Never” to account for the BRFSS questionnaire skip pattern. One final binary question (yes/no) asked: *“Have you or anyone else discussed your confusion or memory loss with a health care professional?”*. All cognitive decline variables were recoded based on BRFSS statistical guidance [11].

### Additional measures and coding

Demographic covariates were sex (Male, Female), age (45-54, 55-64, 65+), race/ethnicity (White, Black, American Indian/Alaskan Native, Asian, Native Hawaiian/Pacific Islander, Other, Multiracial, Hispanic), education (no high school degree, high school graduate, some college education, college graduate or higher), income (<$15K, $15K to <$25K, $25K to <$50K, $50K or more), employment (retired, employed, unemployed), general health (excellent/very good/good, fair/poor), drank any alcoholic beverage in the past 30 days (yes, no), and smoked at least 100 cigarettes in their lifetime (yes, no).

### Analysis

The yearly BRFSS Cognitive Decline data from 2015 to 2020 were combined. Final weights for analysis were recalculated based on CDC guidance to accommodate pooled survey data across multiple years and survey versions [21–24]. All analyses accounted for complex sampling. SAS version 9.4 was used for processing and descriptive statistics. Mplus version 8 was used for the latent class analyses [25]. Latent class analysis was used to predict discrete unobserved groupings (or classes) based on survey item response patterns while accounting for measurement error [26–28]. The relationship between the covariates and categorical latent class outcome were estimated using a logistic link (prediction model). Multinomial logistic regression estimates reflect the likelihood of being part of a particular latent class based on included covariates. To identify the optimal number of latent classes, models are iteratively run increasing the specification of class size from two to five. The optimal number of latent classes is determined through a comparison of model fit statistics and tests, including loglikelihood, Akaike Information Criteria (AIC), Bayesian Information Criteria (BIC), Entropy, Lo-Mendell-Rubin likelihood ratio test (LMR-LRT), and prevalence of latent classes, but most importantly, the conceptual division of subgroups. Entropy ≥ 0.8 indicates classes are sufficiently separated [29]. When comparing model fit statistics, a larger loglikelihood is deemed the best. Alternatively, the smallest AIC and BIC are preferred [30–32]. Lo-Mendell-Rubin Adjusted LRT (LMR-LRT) compares two models with differing number of class specifications. If LMR-LRT has a non-significant test result, then a model with fewer discrete latent classes would fit the data better [33]. Full information maximum likelihood was used in the assessment of latent class models and multinomial logistic regression, which provides unbiased estimates with incomplete/missing data [25]. Predictive modeling included the following covariates: sex, age, race, income, education, employment, general health, drinking behavior, and smoking behavior [25].

## Results

### Descriptive characteristics

Table 1 provides the respondent socio-demographic characteristics and cognitive decline item response frequencies (*n*=54,771). Those experiencing SCD from 2015 to 2020 were 54% female and 41% were 65 years or older. The majority (70%) were non-Hispanic white and had an income of less than $50k (70%). Roughly half (52%) had no more than high school education and were retirees (50%). About half of the respondents reported their health was fair or poor (52%) and smoked at least 100 cigarettes in their lifetime (59%) whereas 40% reported having an alcoholic drink in the past 30 days. As a result of confusion or memory loss, roughly 40% reported giving up day-to-day household activities or chores over the past 12 months “Sometimes”, “Usually”, or “Always”. Half of respondents (49%) reported “Never” needing assistance with day-to-day activities. Subsequently, 13% reported “Always” being able to get the assistance they needed. SCD was reported by 36% to “Sometimes”, “Usually”, or “Always” interfere with one’s ability work, volunteer, or engage in social activities outside of the home. As much as 46% had discussed SCD with a health care professional. In comparison to the excluded respondents without SCD, there was little difference in age, sex, or race/ethnicity. However, 93% of people who reported Excellent/Very Good/Good health did not report having SCD. Compared to those without SCD, those with SCD had a higher proportion of people with an annual household income less than $15K and those with less than a high school education. There was a smaller proportion of those with SCD who were employed compared to those without SCD (data not shown).

**Table 1:** Descriptive statistics of socio-demographics and cognitive decline items, behavioral risk factor surveillance system 2015-2020 (*n*=54,771)

	N^a^	Weighted Percent	95% CI
Sex			
Male	23,670	46.2	(45.1 - 47.3)
Female	31,093	53.8	(52.7 - 54.9)
Age			
45 to 54	10,560	28.1	(27.0 - 29.1)
55 to 64	15,446	30.7	(29.8 - 31.7)
65 or older	28,765	41.2	(40.2 - 42.2)
Race			
White	42,645	69.8	(68.7 - 70.8)
Black	4,536	12.5	(11.8 - 13.2)
American Indian or Alaskan Native	1,085	1.9	(1.6 - 2.1)
Asian	598	2.0	(1.5 - 2.5)
Native Hawaiian or Pacific Islander	95	0.1	(0.1 - 0.2)
Other race	366	0.5	(0.4 - 0.6)
Multiracial	1,307	1.7	(1.5 - 1.9)
Hispanic	3,069	11.6	(10.7 - 12.5)
Education			
Did not graduate High School	6,771	22.2	(21.2 - 23.2)
High School Graduate or GED	16,888	29.7	(28.8 - 30.6)
Some College or Technical School	15,708	29.7	(28.7 - 30.6)
College or Technical School Graduate	15,237	18.4	(17.7 - 19.2)
Income			
<$15,000	8,984	21.2	(20.3 - 22.2)
$15,000-$24,999	11,000	23.5	(22.6 - 24.4)
$25,000-$49,999	11,880	24.9	(23.9 - 25.8)
$50,000+	13,693	30.4	(29.3 - 31.5)
Employment			
Retired	23,947	49.9	(48.7 - 51.1)
Employed	11,789	34.5	(33.3 - 35.7)
Unemployed	4,961	15.6	(14.5 - 16.7)
General Health			
Fair/Poor	26,916	52.1	(51.1 - 53.2)
Excellent/Very Good/Good	27,594	47.9	(46.8 - 48.9)
Drank in past 30 days			
No	32,981	60.3	(59.3 - 61.4)
Yes	21,206	39.7	(38.6 - 40.7)
Smoked at least 100 cigarettes in lifetime			
No	23,179	41.3	(40.3 - 42.4)
Yes	31,277	58.7	(57.6 - 59.7)
Subjective Cognitive Decline Response Items			
*During the past 12 months …*			
As a result of confusion or memory loss, how often have you given up day-to-day household activities or chores you used to do, such as cooking, cleaning, taking medications, driving, or paying bills?			
Always	3,520	7.6	(7.1 - 8.2)
Usually	3,238	6.5	(6.0 - 6.9)
Sometimes	12,717	26.3	(25.4 - 27.3)
Rarely	8,016	14.4	(13.6 - 15.2)
Never	26,255	45.2	(44.1 - 46.2)
As a result of confusion or memory loss, how often do you need assistance with these day-to-day activities?			
Always	3,036	6.8	(6.2 - 7.4)
Usually	2,657	5.2	(4.8 - 5.7)
Sometimes	11,080	22.9	(22.0 - 23.9)
Rarely	8,910	15.9	(15.1 - 16.7)
Never	28,431	49.1	(48.1 - 50.2)
When you need help with these day-to-day activities, how often are you able to get the help that you need?			
Always	6,680	13.4	(12.6 - 14.2)
Usually	3,444	7.0	(6.4 - 7.5)
Sometimes	4,028	9.3	(8.7 - 9.9)
Rarely	1,458	2.9	(2.6 - 3.3)
Never/NA	38,388	67.4	(66.4 - 68.4)
How often has confusion or memory loss interfered with your ability to work, volunteer, or engage in social activities outside the home?			
Always	4,705	9.9	(9.3 - 10.5)
Usually	3,038	6.2	(5.6 - 6.7)
Sometimes	9,814	19.6	(18.8 - 20.5)
Rarely	8,823	16.8	(16.0 - 17.6)
Never	27,307	47.5	(46.4 - 48.6)
Have you or anyone else discussed your confusion or memory loss with a health care professional?			
Yes	25,172	45.7	(44.6 - 46.7)
No	28,870	54.3	(53.3 - 55.4)

Note. *CI* Confidence Interval

^a^ Unweighted frequencies

### Selection of best fitting latent classes

Four latent mixture models were run iteratively increasing the number of latent classes from two to five. Table 2 provides the fit statistics. Considering all evaluated fit statistics, tests, and the conceptual separation of latent classes, the model with three latent classes was optimal. The LMR-LRT was not significant for either the four or five latent class models, indicating that a two or three latent class model was required. Between the two and three latent class model, the Loglikelihood is largest for three classes. Additionally, AIC/BIC are smallest for the three latent class model. While Entropy is higher for the two latent class model, the three class model still provided an ideal value above 0.80 (0.99 vs. 0.85). After the two class model, the Entropy for the three class model was highest of the remaining. The conceptual distinction in response patterns between latent subgroups was intuitive between the three classes, corresponding to a gradient of severity in SCD (“Mild”, “Moderate”, “Severe”).

**Table 2:** Latent class model fit statistics, behavioral risk factor surveillance system 2015-2020 (*n*=54,771)

# Classes^a^	Loglikelihood^a^	AIC^b^	BIC^b^	Entropy^c^	LMR-LRT^d^
2	-262390	524851	525163	0.99	*p* < 0.05
3	-255850	511806	512279	0.85	*p* < 0.05
4	-251850	503841	504474	0.83	*p* = 0.30
5	-250544	501266	502059	0.81	*p* = 0.55

^a^ Larger values represent better fitting models

^b^ Smaller values represent better fitting models

^c^ Desired entropy above 0.80

^d^ Non-significant values (α=0.05) indicate models of fewer latent classes are better fitting

### Latent class analysis

The selected model separates response patterns into three discrete latent classes. Conditional and unconditional probability estimates from the 3-class model appear in Table 3. Conditional response probabilities differentiate latent subgroups by severity of SCD. Thus, the three classes are best interpreted as *Mild*, *Moderate*, and *Severe* SCD. The first class is the largest (42.9%), representing those who report mild SCD. Respondents in this class had a very low probability of giving up on or needing assistance with household chores or daily activities due to SCD. They did not have trouble getting help for daily activities or chores because it was unnecessary. There was a high probability that SCD never effected one’s ability to participate in social activities. And, members of this class had a 71.2% probability to have never had discussions with a healthcare professional about their experiences of SCD. The second, and smallest (22.3%), class represented the latent subgroup for those with moderate SCD. Members of this group were more likely to “Rarely” or “Sometimes” give up on day-to-day activities due to SCD. Further, the Moderate group were more likely to “Rarely” or “Never” require assistance for these day-to-day activities. Although, 26.7% would sometimes find SCD to interfere with work, volunteering, or social activities, and 53.4% spoke to a health professional regarding the issue. The third class contained 34.8% of the sample, representing those who have severe SCD. These respondents had a high probability of occasionally giving up household chores or daily activities due to SCD and often need assistance. Members of the Severe class occasionally feel SCD interferes in social situations and 61.8% discussed SCD with a healthcare professional.

**Table 3:** Latent class conditional response probabilities^a^, behavioral risk factor surveillance system 2015-2020 (*n*=54,771)

	Subjective Cognitive Decline^b^		
	Mild	Moderate	Severe
	42.9%^c^	22.3%^c^	34.8%^c^
*During the past 12 months …*			
As a result of confusion or memory loss, how often have you given up day-to-day household activities or chores you used to do, such as cooking, cleaning, taking medications, driving, or paying bills?			
Always	1.4%	4.3%	17.6%
Usually	0.6%	5.5%	14.4%
Sometimes	5.5%	35.6%	46.3%
Rarely	9.8%	32.5%	8.4%
Never	82.7%	22.1%	13.4%
As a result of confusion or memory loss, how often do you need assistance with these day-to-day activities?			
Always	0.1%	0.0%	19.5%
Usually	0.1%	0.0%	15.0%
Sometimes	0.3%	0.3%	65.4%
Rarely	6.6%	58.6%	0.0%
Never	93.0%	41.0%	0.0%
When you need help with these day-to-day activities, how often are you able to get the help that you need?			
Always	0.0%	0.0%	38.8%
Usually	0.0%	0.0%	20.2%
Sometimes	0.0%	0.0%	26.8%
Rarely	0.0%	0.0%	8.5%
Never/NA	100.0%	100.0%	5.7%
How often has confusion or memory loss interfered with your ability to work, volunteer, or engage in social activities outside the home?			
Always	0.4%	7.0%	23.5%
Usually	0.0%	5.9%	14.0%
Sometimes	4.2%	26.7%	34.3%
Rarely	9.4%	38.0%	12.4%
Never	85.9%	22.3%	15.8%
Have you or anyone else discussed your confusion or memory loss with a health care professional?			
Yes	28.8%	53.4%	61.8%
No	71.2%	46.6%	38.2%

^a^ Conditional response probabilities (0-100) represent the probability of selecting a response option based on a respondent's latent class membership. For example, among the subgroup of respondents who have *mild* SCD, the probability of selecting "Always" giving up day-to-day household activities or chores is low at 1.4%

^b^ The latent subgroups represent levels of severity for SCD. Respondents who have mild SCD can be interpreted as having a higher probability of selecting a response option related to sparse experiences, such as "Rarely" or "Never". The Moderate subgroup has a slightly probability of selecting "Never" but a higher probability of choosing "Rarely" or "Sometimes". Alternatively, the Severe subgroup have a lower probability of selecting the same response options and a much higher probability of choosing "Usually" or "Always" experiencing SCD

^c^ Unconditional probability. Proportion of sample who fall into each latent class

### Prediction model

The results of the adjusted multinomial logistic regression are shown in Table 4. Compared to the Mild subgroup, Moderate SCD are significantly less likely to be older (65+ vs 45-54: OR = 0.47, 95% CI: 0.38-0.58), employed (employed vs. retired: OR = 0.78, 95% CI: 0.65-0.94), report good, very good, or excellent general health (excellent/very good/good vs. fair/poor: OR = 0.49, 95% CI: 0.44-0.54), and to have had a drink in the past 30 days (drank alcohol vs. did not drink alcohol: OR = 0.85, 95% CI: 0.75-0.97). Further, members were significantly more likely to have an income below $50k a year (<$15K vs. $50k+: OR = 2.22, 95% CI: 1.78-2.77), be unemployed (unemployed vs. retired: OR = 1.27, 95% CI: 1.01-1.60), or smoked 100 cigarettes in their lifetime (smoked vs. did not smoke: OR = 1.15, 95% CI: 1.03-1.28). The effects for the Severe subgroup follow the same trends but demonstrate an even stronger relationship and additionally significant predictors. For example, compared to the Mild subgroup, the Severe SCD were less likely to be Female (Female vs. Male: OR = 0.85, 95% CI: 0.74-0.96) but more likely to be Black, Multiracial, or Hispanic (non-Hispanic Black vs. non-Hispanic White: OR = 1.80, 95% CI: 1.53-2.11; non-Hispanic Multiracial vs. non-Hispanic White: OR = 1.42, 95% CI: 1.04-1.94; Hispanic vs. non-Hispanic White: OR = 1.68, 95% CI: 1.40-2.03), and have less than a college or technical degree (<high school vs. college/tech school graduate: OR = 1.60, 95% CI: 1.34-1.91).

**Table 4:** Prediction of latent class membership by socio-demographics, behavioral risk factor surveillance system 2015-2020 (*n*=54,771)

	Subjective Cognitive Decline (ref=Mild)			
	Moderate^a^		Severe^a^	
	OR	95% CI	OR	95% CI
Sex				
Male	ref		ref	
Female	1.05	(0.91-1.21)	**0.85**	**(0.74-0.96)**
Age				
45-54	ref		ref	
55-64	**0.78**	**(0.66-0.92)**	**0.64**	**(0.54-0.77)**
65+	**0.47**	**(0.38-0.58)**	**0.29**	**(0.23-0.35)**
Race				
White	ref		ref	
Black	1.02	(0.83-1.26)	**1.80**	**(1.53-2.11)**
American Indian/Alaskan Native	1.23	(0.82-1.85)	1.49	(0.96-2.32)
Asian	1.35	(0.63-2.88)	1.32	(0.74-2.33)
Native Hawaiian/Pacific Islander	0.92	(0.25-3.40)	1.50	(0.46-4.94)
Other	1.10	(0.68-1.78)	1.59	(0.91-2.79)
Multiracial	1.18	(0.72-1.92)	**1.42**	**(1.04-1.94)**
Hispanic	1.26	(0.99-1.60)	**1.68**	**(1.40-2.03)**
Education				
Did not graduate High School	1.02	(0.83-1.27)	**1.60**	**(1.34-1.91)**
High School Graduate or GED	1.00	(0.83-1.19)	**1.23**	**(1.05-1.45)**
Some College or Technical School	1.13	(0.95-1.33)	**1.27**	**(1.08-1.49)**
College or Technical School Graduate	ref		ref	
Income				
<$15,000	**2.22**	**(1.78-2.77)**	**3.03**	**(2.43-3.77)**
$15,000-$24,999	**2.07**	**(1.71-2.50)**	**2.19**	**(1.85-2.59)**
$25,000-$49,999	**1.61**	**(1.39-1.87)**	**1.49**	**(1.20-1.85)**
$50,000+	ref		ref	
Employment				
Retired	ref		ref	
Employed	**0.78**	**(0.65-0.94)**	**0.42**	**(0.34-0.53)**
Unemployed	**1.27**	**(1.01-1.60)**	0.95	(0.78-1.16)
General Health				
Fair/Poor	ref		ref	
Excellent/Very Good/Good	**0.49**	**(0.44-0.54)**	**0.31**	**(0.28-0.34)**
Drank in past 30 days				
No	ref		ref	
Yes	**0.85**	**(0.75-0.97)**	**0.59**	**(0.53-0.66)**
Smoked at least 100 cigarettes in lifetime				
No	ref		ref	
Yes	**1.15**	**(1.03-1.28)**	1.07	(0.96-1.18)

*Note*. *OR* Odds Ratio, *CI* Confidence Interval. Bold font represents significant findings at α = 0.05. All variables are adjusted for the other presented covariates

## Discussion

The purpose of this study was to investigate latent subgroups of SCD severity in the last year using BRFSS 2015-2020 and to identify associations with group membership by socio-demographic covariates. The analysis provided reasonable empirical evidence to support the hypothesis that discrete latent subgroups of SCD exist. Specifically, three discrete latent classes – Mild, Moderate, and Severe – represented indicators of SCD. To the best of our knowledge, this is the first study to use LCA to distinguish unobserved subgroups of SCD and demonstrate evidence of associated socio-demographic characteristics. Regarding Moderate SCD, age (65+ vs. 45-54; 55-64 vs. 45-54), employment (employed vs. retired), general health (excellent/very good/good vs. fair/poor), and having had a drink in the past 30 days are significantly *negatively* associated. Whereas income (<$15K vs. $50K+; $15-24K vs. $50K+; $25-49K vs. $50K+), unemployment (unemployed vs. retired), and having smoked at least 100 cigarettes in their lifetime are significantly *positively* associated with Moderate SCD. Regarding Severe SCD, race (non-Hispanic Black vs. non-Hispanic White; non-Hispanic Multiracial vs. non-Hispanic White; Hispanic vs. non-Hispanic White), education (<high school vs. college/tech school graduate; high school grad vs. college/tech school graduate; some college/tech school vs. college/tech school graduate), and income (<$15K vs. $50K+; $15-24K vs. $50K+; $25-49K vs. $50K+) are significantly *positively* associated. However, age (65+ vs. 45-54; 55-64 vs. 45-54), employment (employed vs. retired), general health (excellent/very good/good vs. fair/poor), and having had a drink in the past 30 days are significantly *negatively* associated with Severe SCD.

To the best of the authors’ knowledge, this is the first study to use a self-reported questionnaire to assess subgroups of cognitive impairment, which has utility for understanding the severity of SCD and implications for public health. This study extends the use of the SCD module for population-based surveillance of cognitive functioning. For example, indicators of SCD severity could advance surveillance as recommended in M-3 of the 3rd Edition: “State and Local Public Health Partnerships to Address Dementia: The 2018-2023 Road Map [9]. Understanding variability of SCD would improve program planning and resource allocation for state health systems and begin to address societal factors related to the burden of disease. Subsequently, one of the goals of the initiative is to diminish inequalities of Alzheimer's disease and related dementias in consideration of social determinants of health. Similar to prior research, our findings show disproportionate SCD based on race/ethnicity and socio-economic indicators (e.g., income and education) [34, 35]. However, we not only identify these characteristics as predictors of SCD but demonstrate that people who have low household income or educational attainment are most likely to have the worst symptoms. Discerning the nuances of SCD severity allows for improved tailoring of public health measures for the most affected communities. Furthermore, this study elucidates important findings regarding communication of SCD with a healthcare professional. Specifically, SCD severity is related to talking with a healthcare provider. The probability of discussing symptoms with a provider was over 50% for both the Moderate (53.4%) and Severe (61.8%) SCD groups, while only 28.8% for the Mild group. These findings not only provide further support for the Healthy People 2030 objective (DIA-03), but also identify target populations to improve the metric (i.e., “*increase the proportion of adults with subjective cognitive decline who have discussed their symptoms with a provider”)*. All groups need to improve the proportion who have discussed symptoms with a provider. In particular, public health efforts should focus on the mild group with only roughly a quarter of these adults likely to speak with a healthcare professional. Even at the mild stage, early detection poses a great benefit to the affected individuals, caregivers, and overall healthcare costs [36]. Communication with a provider allows adults to eliminate other sources of dementia-like symptoms. If mild SCD develops into dementia, an early diagnosis gives patients more time to get symptomatic treatments, potentially enroll in clinical trials, as well as make legal and care arrangements with family. To improve this metric, public health efforts should be made to reduce the stigma surrounding discussing symptoms with family members, their healthcare providers, and to bring awareness for the need to have these discussions [37, 38].

Previous studies benefitted from the ability to assess cognitive profiles; however, many available assessments of cognition can be prohibitive for population surveillance due to the amount of resources necessary. The use of the cognitive decline module in tandem with the results of this study expands the utility of surveillance. Additionally, the current study includes an expanded list of socio-demographic covariates to prior research (i.e., age, sex, and education) which provides greater public health context to social and behavioral disparities associated with cognitive decline [15, 17, 39–41]. Among the two studies which used the prediction model to assess associations with group membership, our study found similar results for education yet disparate findings for age and sex [15, 40]. In contrast to our study, each found sex to be significantly related to latent class membership [15, 40]. These studies found males had significant relationships with cognitive profiles (positive: attention/ construction symptoms; negative: memory symptoms) whereas females were significantly positively associated mild to severe impairment generally [15, 40]. Considering these findings, there may remain differences in cognitive profiles within our latent subgroups. Separately, Davidson et al. (2010) and Scheltens et al. (2016) both found low education to be significantly associated with greater severity of Alzheimer’s. SCD is frequently a feature of Alzheimer’s disease and these results contribute to the evidence that educational attainment is an important modifiable risk factor for prevention of future cognitive impairment and disease progression. Lastly, contrary to Davidson’s findings, our study showed that younger age is associated with moderate and severe SCD [40]. While opposing the logical expectation, one reason for members of the Moderate or Severe SCD groups to more likely be younger could be due to stigma of failing cognition affecting the way older respondents report the frequency of SCD [38, 42]. However, it is also possible that respondents who have a higher probability of developing Alzheimer’s disease or a dementia-related disease, such as those with a lower educational attainment, are experiencing cognitive decline at a younger age [43, 44].

There are limitations to this study that should be considered. First, our sample only includes community-dwelling individuals and may exclude those with limitations associated with cognitive functioning. Second, coding respondents who reported never or rarely needing assistance with day-to-day activities (*n*=37,341, 68%) as “never” for item 4 could affect the probabilities for the Never/NA response. A sensitivity analysis demonstrated similar LCA model fit results with and without imputation. Final model selection was not affected. Third, when interpreting the results of this analysis, only respondents who experienced SCD were included. In post hoc analyses, the model was tested on the full sample as well. Restricting the sample to those with SCD improved model fit and conceptual distinction of classes. There are strengths to this study. First, using latent class analysis is superior to other common methods accounting for measurement error to improve precision of estimates [45]. And, second, the previous studies yield prudent findings through diagnostic means but are limited by cost and logistical feasibility for population-level surveillance. Specifically, there is practical use for the three latent indicators of severity from our findings. Rather than continuing as item-by-item analysis of this module, latent classes more easily quantify the severity of SCD. Future research is necessary to expand this work. For example, research should investigate the neurological differences between members of the Mild, Moderate, and Severe SCD groups.

In summary, latent class analysis has useful applications for population-level surveillance measures. This study demonstrated respondents of the BRFSS’ cognitive decline module cluster into three discrete latent subgroups regarding the severity of SCD (Mild, Moderate, and Severe). Socio-demographics were associated with membership in each group. Although the cognitive decline module is not a diagnostic tool, using these discrete latent groups can provide clarity for SCD prevalence in the population. Specifically, the use of the three latent subgroups, rather than item-by-item analysis, allows for a more intuitive understanding of the public health burden. These study findings could easily be utilized in an applied or academic setting. For example, opposed to assessing discrete items, a holistic approach to more fully understand the national and state-level epidemiology of SCD is now possible. While individual items provide important information regarding SCD, the overall health burden has historically been more challenging to interpret. Using this new LCA indicator, researchers can easily quantify SCD severity nationally or within their locale. Further, this method allows for facile identification of respondent characteristics based on SCD severity, and it can also be used to consider spatial distribution in various geographies. Considering the progression from mild cognitive impairment to dementia-related illnesses, it is particularly important for public health to continue to improve the ability to monitor the aging health of our population and it’s effect on our communities [46]. The labor demand of caregivers for ADRD adults has negative impacts on physical and mental health. Additionally, caregivers are insufficiently remunerated with a share of roughly 20 hours per week per caregiver being unpaid [36]. Further, the direct cost of Alzheimer’s disease in 2019 is $290 billion with 67% covered by Medicare and Medicaid [36]. Through improved cognitive decline surveillance and response, there is potential to stymie the substantial societal and financial impact of Alzheimer’s disease and dementia-related disorders.

## Data Availability

The dataset generated and analyzed during the current study is made available by the Centers for Disease Control and Prevention and accessed through the annual survey data and documentation page of the Behavioral Risk Factor Surveillance System, https://www.cdc.gov/brfss/annual_data/annual_data.htm.
